# Beyond hostility: exploring facial emotion recognition biases in youths with conduct disorder

**DOI:** 10.1007/s00787-025-02846-y

**Published:** 2025-08-27

**Authors:** Janine Bacher, Beryll von Planta, Anka Bernhard, Graeme Fairchild, Lucres Jansen, Stephane A. De Brito, Christine M. Freitag, Kerstin Konrad, Christina Stadler, Gregor Kohls, Eva Unternaehrer

**Affiliations:** 1 Child and Adolescent Psychiatric Research Department, University Psychiatric Clinics Basel, Basel, Switzerland; 2 Department of Child and Adolescent Psychiatry, Medical Faculty, German Center for Child and Adolescent Health (DZKJ), TUD Dresden University of Technology, partner site Leipzig/Dresden, Dresden, Germany; 3 Department of Child and Adolescent Psychiatry, Psychosomatics and Psychotherapy, University Hospital Frankfurt, Goethe University, Frankfurt am Main, Germany; 4 Department of Psychology, University of Bath, Bath, UK; 5 Department of Child and Adolescent Psychiatry, VU University Medical Center, Amsterdam, the Netherlands; 6 Centre for Human Brain Health, School of Psychology, University of Birmingham, Birmingham, UK; 7 Child Neuropsychology Section, Department of Child and Adolescent Psychiatry, Psychosomatics and Psychotherapy, RWTH Aachen University, Aachen, Germany; 8 JARA-Brain Institute II, Molecular Neuroscience and Neuroimaging, RWTH Aachen & Research Centre Juelich, Juelich, Germany

**Keywords:** Emotion recognition bias, Facial emotion recognition, Conduct disorder, Antisocial behavior, Adolescent, FemNAT-CD

## Abstract

Facial emotion recognition (FER) biases refer to systematic tendencies to recognize specific emotions when processing facial expressions. In youths with conduct disorder (CD), who are characterized by highly impairing antisocial behavior, research on FER biases has focused on hostile attribution biases. This work has shown that youths with CD perceive ambiguous social cues as angry. However, youths with CD may not only show biases towards anger, which is why we investigated FER biases in youths with CD towards the six basic emotions. Within the European FemNAT-CD study, we analyzed data from 610 youths with CD (60% female) and 818 typically developing controls (TDCs; 68% female), aged 9 to 18 years (*M* = 14.1, *SD* = 2.41 years). FER biases were assessed using the Emotion Hexagon Task by showing morphed emotional expressions and asking participants to choose the predominant emotion. Biases were calculated as tendency towards an emotion shown at 0%, 10%, 30%, or 50% intensity. Our findings from hierarchical linear modelling indicate that youths with CD exhibited stronger FER biases than TDCs across all emotions, meaning that they misclassified each emotion more often. However, this difference varied by intensity, with youths with CD displaying weaker biases at higher intensity levels and a smaller increase in bias with increasing intensity level. Our findings indicate that youths with CD not only show a hostile attribution bias but rather misclassify emotions as predominant when they are present at low intensity, regardless of type of emotion.

**Supplementary Information:**

The online version contains supplementary material available at 10.1007/s00787-025-02846-y.

## Introduction

Youths with conduct disorder (CD) show highly impairing patterns of aggressive and antisocial behavior [1]. They also display impairments in the ability to recognize facial expressions [2], which is crucial for understanding social interactions [3]. Facial expressions serve as nonverbal cues communicating emotional states and intentions, influencing how others respond [4]. Being able to identify these expressions supports accurate interpretation of social contexts and improves interpersonal interactions [5].

Previous studies investigating facial emotion recognition (FER) impairments in youths with CD have primarily focused on FER accuracy, that is, whether emotions are correctly identified [e.g., 6]. Rather than focusing solely on FER accuracy, biases in FER may, however, be more predictive of antisocial behavior [6]. Biases in FER can be defined as systematic tendencies towards recognizing specific emotions when processing facial expressions [7], which can lead to distorted social information processing and maladaptive behavioral responses [6]. This distinction is conceptually important, as categorization accuracy alone does not reveal what emotion is chosen instead, which is a key piece of information when trying to understand emotion recognition impairments in CD. Youths with CD might show a tendency towards perceiving ambiguous emotional expressions as angry (i.e., anger bias) [8]. Based on the social information-processing theory proposed by Dodge and Crick [9], this in return may cause them to respond aggressively (i.e., with anger) and increase the likelihood of being rejected by peers due to their aggressive behavior [10]. Youths with CD might also feel rejected if they show a tendency towards perceiving an ambiguous emotional expression as disgust (i.e., disgust bias), since perceiving facial expressions of disgust can trigger increased shame [11].

Importantly, FER biases are not the same as attributional biases, which are grounded in attribution theory [12]. They refer to how individuals explain the causes of others’ behaviors [13] and have been studied in youths with CD in terms of a hostile attribution biases [14]. FER biases also differ from attentional biases, which describe the tendency to pay more attention to certain emotional stimuli over others and show difficulties disengaging from them [15]. Our construct of FER biases specifically refers to systematic tendencies towards specific emotions when processing and labelling facial expressions, without involving explanations of behaviors or the duration of attention towards emotional stimuli. Given this distinction, the present study concentrates on examining FER biases independently from attributional or attentional biases.

A central question is for which emotions youths with CD show a FER bias. In a study investigating FER, youths with conduct problems aged 7 to 13 years showed a bias for the emotion anger when labelling facial expressions [8]. In contrast, Ciucci and colleagues [16] found no association between conduct problems in youths aged 11 to 14 years and FER bias towards anger. The scale they used in their study to assess conduct problems was reported to correlate highly with independent clinical diagnoses of conduct disorder [17]. But research so far has not only recognized an anger bias in youths with CD: Short and colleagues [18] found that youths with CD aged 12 to 18 years were more likely to classify neutral faces as fearful and therefore showed a fear bias rather than an anger bias. In related conditions like general disruptive behavior, youths between the ages of 8 and 18 showed FER biases for anger, happiness and surprise depending on aggression subtype and callous-unemotional traits [19]. Taken together, previous findings on FER biases in youths with CD are inconsistent, suggesting that biases may not be limited to anger but could also involve fear, happiness, or surprise.

The aim of this study was to examine FER biases in youths with CD for all six basic emotions compared to typically developing controls (TDCs) across different intensity levels, since analyzing FER biases across emotion intensities may offer deeper insights into the emotional processing mechanisms underlying antisocial behavior in youths with CD [20]. Overall, we expected differences in FER biases between youths with CD and TDCs based on prior research on youths with CD or related conditions [8, 18, 19]. We also expected that these differences would vary depending on emotion and intensity level. However, we investigated these FER biases in bi-directional hypotheses due to the lack of previous evidence.

## Methods

### Participants and procedure

This study included 1,428 participants from the European multi-site project *Neurobiology and Treatment of Adolescent Female Conduct Disorder: The Central Role of Emotion Processing* (FemNAT-CD) who completed the Emotion Hexagon Task and fulfilled our data quality criteria (see Online Resource 1). The sample consisted of 610 (60% female) youths diagnosed with CD and 818 (68% female) TDCs from Germany, the United Kingdom, Spain, the Netherlands, Greece, Switzerland, and Hungary. Participants were aged 9 to 18 years (*M* = 14.1, *SD* = 2.41 years), and estimated full-scale IQ ranged from 70 to 145 (*M* = 100.1, *SD* = 12.6). They were recruited through clinical referrals, youth offending services, schools, and community channels (e.g., social media). Inclusion criteria for the CD group consisted of a current CD diagnosis according to DSM-IV-TR criteria [21]. Participants taking psychotropic medication were assessed while remaining on their prescribed medication (e.g., psychostimulants). Overall exclusion criteria were an IQ below 70, diagnosis of autism spectrum disorders, schizophrenia, bipolar disorder or mania, neurological disorders or genetic syndromes (e.g., Down Syndrome). For the TDCs group, exclusion criteria were any current DSM-IV-TR diagnosis or a history of CD, ODD, or ADHD.

### Measures

#### Facial emotion recognition biases

FER biases were assessed using the Emotion Hexagon Task, which consists of identifying facial expressions based on the Ekman-Friesen Pictures of Facial Affect [22, 23]. Each stimulus is a computer-generated blend (morph) of two emotions at varying intensities (e.g., 90% anger and 10% disgust). After a practice block, participants completed five blocks, each containing 30 trials. In each trial, participants viewed a facial expression for 3 s and were asked to state which emotion best described the expression shown. They could choose between labels for the six basic emotions happiness, surprise, fear, sadness, disgust and anger. Blends included 10%:90%, 30%:70% and 50%:50% intensities. The specific emotion combinations were based on confusability norms established by Ekman and Friesen [23], with each emotion placed adjacent to the one it was most frequently confused with [22]. By connecting the ends of the sequence to form a hexagon, six emotion continua were created for the Emotion Hexagon Task by morphing images along each edge: happiness–surprise, surprise–fear, fear–sadness, sadness–disgust, disgust–anger, and anger–happiness. E-Prime was used to implement the task and present the stimuli in random order.

In the present study, FER biases were calculated for each of the six basic emotions and computed across four levels of emotional expression intensity (0%, 10%, 30%, and 50%). To account for expressions with maximum ambiguity, in accordance with Schönenberg and Jusyte [24], we also included as bias when the indicated emotion was represented at 50% intensity in a blend, as such bias would still reflect a tendency towards a specific emotion. The computation procedure was identical for all FER biases; for illustrative purposes, the process is described in detail below using the example of the anger bias (Table 1). The dataset was first filtered to include only trials where participants selected *anger* as the emotion that best described the expression shown. In this filtered dataset, for the 0% intensity level, all trials were included where anger was not present in the stimulus (i.e., combinations of happiness–surprise, surprise–fear, fear–sadness, and sadness–disgust). For 10%, 30%, and 50% intensity levels, trials were included where *anger* was blended with either of its two neighboring emotions (*happiness* or *disgust*) at the respective proportions (e.g., 10% anger : 90% happiness, or 10% anger : 90% disgust). Therefore, the 0% intensity level biases included a maximum of 100 possible trials, while each of the other intensity levels included up to 10 trials. To account for this difference, we calculated percentage bias scores by dividing the number of *anger* responses by the total possible number of trials at each intensity level and multiplying by 100. Final bias scores were then computed as the mean percentage bias across all intensity levels for each emotion.

**Table 1:** Calculation of FER biases on the example of the anger bias

	Intensity Level			
Emotion Bias	0%	10%	30%	50%
Anger	All other combinations: Happiness : Surprise ^b^ Surprise : Fear ^b^ Fear : Sadness ^b^ Sadness : Disgust ^b^	10% Anger : 90% Happiness ^a^ 10% Anger : 90% Disgust ^a^	30% Anger : 70% Happiness ^a^ 30% Anger : 70% Disgust ^a^	50% Anger : 50% Happiness ^a^ 50% Anger : 50% Disgust ^a^

^a^5 trials

^b^25 trials

#### Demographic variables

In addition to age and biological sex, cognitive abilities (IQ) and socioeconomic status (SES) were assessed. IQ was measured using the vocabulary and matrix reasoning subtests of the Wechsler Intelligence Scale (WISC-III-R/WISC-IV for adolescents up to the age of 16, WAIS-III-R/WAIS-IV for adolescents aged 17–18) or the Wechsler Abbreviated Scale of Intelligence (WASI) at UK sites [25–28]. SES was scored based on the Medical History Questionnaire; a semi-structured interview specifically designed for this study which was used to gather data regarding the medical history of participant [29]. The interview included questions about parental income, education level and occupation and was used to score SES by principal component extraction standardized per country. To assess parental educational status, ISCED-classifications were used [30].

#### Statistical analyses

All analyses were conducted using R Statistical Software version 4.4.2 [31] for Windows (for details of used packages see Online Resource 1). We tested for demographic differences between groups using chi-square tests for categorial variables (age, sex) and t-tests for continuous variables (IQ, SES). For all analyses, p-values below 0.05 were considered statistically significant. Multilevel models (MLMs) were used to account for dependent observations within participants. On the person level, we included the effect of group to explore group differences in FER biases between youths with CD and TDCs and controlled for age, sex, IQ and SES. On the observation level, we added emotion type as factor and intensity as a continuous variable to see whether these influenced FER biases. MLMs account for multiple comparisons through partial pooling, meaning that person- or observation-level estimates are “shrunk” toward the overall mean, which reduces the risk of false positives [32]. To support the validity of this approach, we report model fit comparisons of alternative models (see ‘Preliminary findings – model fit’) [33]. We gradually added effects on both levels and compared the model fits using Akaike Information Criterion (AIC), Bayesian Information Criterion (BIC), and the Maximum Likelihood Ratio Test. We also calculated the marginal R^2^ to estimate effect sizes.

## Results

### Descriptive statistics

The demographic variables per group and comorbidities of the CD group are presented in Table 2. There was a significant group difference in age, with youths with CD being older than TDCs, and sex, with the CD group having a lower proportion of female participants. The groups significantly differed in IQ, with youths with CD having lower IQs than TDCs. Youths with CD also had a significantly lower SES than TDCs.

**Table 2:** Descriptive statistics of demographic variables and comorbidities

	CD *n* = 610	TDCs *n* = 818	Group effect *p*-values ^a^
Age (years) *M* (SD)	14.29 (2.3)	13.97 (2.47)	< 0.05
Females *n* (%)	365 (59.8)	553 (67.6)	< 0.01
IQ *M* (SD)	95.7 (11.87)	103.39 (12.17)	< 0.01
SES *M* (SD) ^b^	− 0.29 (0.95)	0.29 (1.01)	< 0.01
Major Depressive Disorder *n* (%)	85 (13.93)		
Attention Deficit Disorder *n* (%)	224 (36.72)		
Posttraumatic Stress Disorder *n* (%)	45 (7.38)		
Oppositional Defiant Disorder *n* (%)	491 (80.49)		
Generalized Anxiety Disorder *n* (%)	20 (3.28)		
Social Phobia *n* (%)	26 (4.26)		
Psychotropic Medication *n* (%)	190 (31.15)		

^a^ *p*-values are based on two-sample t-tests or χ^2^ tests

^b^ principal component extracted from socioeconomic status standardized by country (M = 0, SD = 1)

*CD *Conduct Disorder, *TDCs *typically developing controls

### Multilevel analysis

#### Preliminary findings – model fit

We hierarchically built multilevel models and compared model fit to find the best-fitting solution (see Online Resource; Supplementary Table 1). To ensure transparent model selection, we detail this process by reporting the fit comparisons of several alternative models. First, a baseline model (null model) was fitted, where FER biases were predicted by a fixed intercept. Then, a random intercept model was tested to account for clustering of observations within participants. However, a model comparison suggested that the random intercept did not significantly improve model fit compared to a fixed intercept model. Despite this, we continued with the random intercept model because of structural reasons as trials were nested within participants to account for possible dependency in the data and to allow for further model extensions. Several model comparisons showed that the final model included the fixed effect of type of emotion, a quadratic fixed effect of intensity of emotion, group, as well as the interaction terms group * emotion, group * intensity, the three-way interaction term group * emotion * intensity and the covariates age and sex. Adding IQ and SES as control variables did not improve model fit, neither did including group * age, group * sex or group * IQ as interaction terms. Marginal R^2^ for the final model including all significant predictors, covariates and interactions was 0.52, meaning that the fixed effects in the model explained 52% of the variance in FER biases.

#### Main findings – final model

**Fig. 1:**
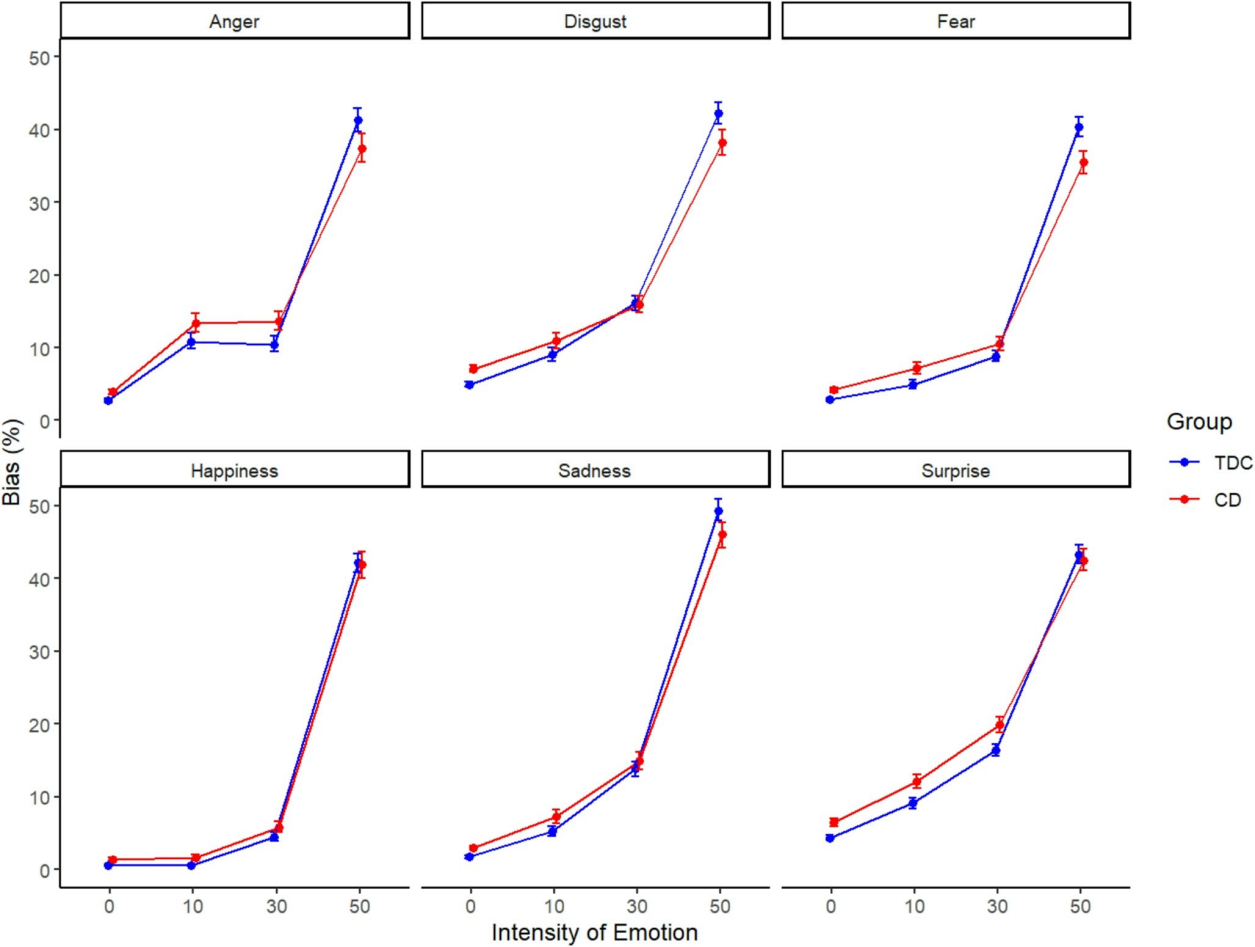
Facial emotion recognition biases for each emotion and intensity level by group

The final model revealed that the CD group had overall significantly stronger FER biases than TDCs (Table 3). There was no significant interaction between group and type of emotion, indicating that group differences in FER biases did not vary systematically across emotions. However, there was a significant interaction between group and intensity, indicating that the strength of FER biases across intensity levels differed between groups (Fig. 1).

Specifically, youths with CD showed stronger FER biases than TDCs at lower intensity levels, but a smaller increase with increasing intensity level. This pattern is indicated by the significant negative quadratic effect. The three-way interaction between group, type of emotion and intensity level further revealed that this difference in FER biases across intensity levels between groups also differed between emotions. We demonstrate this complex interaction by comparing two example emotions. For the emotion fear, the difference between groups at the 50% intensity level was greater than for the emotion surprise, whereas at the 30% intensity level, the difference between groups in FER biases was smaller for fear than for surprise. Overall, the pattern of intensity-related changes in FER biases differed not only between groups but also across emotions.

**Table 3:** Final multilevel model: results predicting FER biases

	B	SE		df	t	*p*
(Intercept)	19.4		0.52	32,810	37.22	< 0.001
Type of Emotion						
Anger	Reference					
Disgust	1.78		0.35	32,810	5.07	< 0.001
Fear	−2.08		0.35	32,810	−5.9	< 0.001
Happiness	−4.39		0.35	32,810	−12.48	< 0.001
Sadness	1.21		0.35	32,810	3.43	< 0.001
Surprise	1.94		0.79	32,810	5.5	< 0.001
Intensity						
Linear	2451.13		46.1	32,810	53.17	< 0.001
Quadratic	956.98		46.1	32,810	20.76	< 0.001
Age	−0.25		0.03	1,424	−7.5	< 0.001
Male (vs. Female)	1.01		0.16	1,424	6.25	< 0.001
Group CD (vs. TDCs)	0.77		0.38	1,424	2.01	0.04
Group * Emotion						
CD * Anger	Reference					
CD * Disgust	−0.84		0.54	32,810	−1.56	0.12
CD * Fear	−0.65		0.54	32,810	−1.2	0.23
CD * Happiness	−0.02		0.54	32,810	−0.04	0.97
CD * Sadness	−0.51		0.54	32,810	−0.95	0.34
CD * Surprise	1.2		0.54	32,810	2.22	0.03
Group * Intensity						
CD * Linear	−342.07		70.53	32,810	−4.85	< 0.001
CD * Quadratic	−378.2		70.53	32,810	−5.36	< 0.001
Group * Intensity * Emotion						
TDCs * Linear * Anger	Reference					
TDCs * Linear * Disgust	103.26		65.2	32,810	1.58	0.11
TDCs * Linear * Fear	82.74		65.2	32,810	1.27	0.2
TDCs * Linear * Happiness	377.81		65.2	32,810	5.8	< 0.001
TDCs * Linear * Sadness	817.86		65.2	32,810	12.54	< 0.001
TDCs * Linear * Surprise	207.74		65.2	32,810	3.19	0.001
TDCs * Quadratic * Anger	Reference					
TDCs * Quadratic * Disgust	−171.74		65.2	31,382	−2.63	0.008
TDCs * Quadratic * Fear	221.81		65.2	31,382	3.4	< 0.001
TDCs * Quadratic * Happiness	582.03		65.2	31,382	8.93	< 0.001
TDCs * Quadratic * Sadness	233.07		65.2	31,382	3.57	< 0.001
TDCs * Quadratic * Surprise	−161.29		65.2	31,382	−2.47	0.01
CD * Linear * Anger	Reference					
CD * Linear * Disgust	3.97		75.5	31,382	0.05	0.96
CD * Linear * Fear	−4.28		75.5	31,382	−0.06	0.95
CD * Linear * Happiness	652.16		75.5	31,382	8.64	< 0.001
CD * Linear * Sadness	832.73		75.5	31,382	11.03	< 0.001
CD * Linear * Surprise	354.75		75.5	31,382	4.7	< 0.001
CD * Quadratic * Anger	Reference					
CD * Quadratic * Disgust	97.91		75.5	31,382	1.3	0.19
CD * Quadratic * Fear	290.55		75.5	31,382	3.85	< 0.001
CD * Quadratic * Happiness	878.13		75.5	31,382	11.63	< 0.001
CD * Quadratic * Sadness	413.22		75.5	31,382	5.47	< 0.001
CD * Quadratic * Surprise	−26.56		75.5	31,382	−0.35	0.72

*CD* Conduct Disorder, *TDCs* typically developing controls, *B* unstandardized beta, *SE* standard error, *df* degrees of freedom, *t* t-statistic

## Discussion

The most commonly studied aspect of FER biases in youths with CD is the anger bias, whereby ambiguous facial expressions are misinterpreted as displaying anger, which could be perceived as a threat that potentially leads to aggression [8]. However, FER biases in youths with CD might be broader beyond a bias towards anger (e.g., bias towards fear or happiness) [18, 19] and depend on the intensity level of the presented facial expression. This study therefore expands the current knowledge base by examining FER biases in youths with CD compared to TDCs for all six basic emotions considering both type and different intensity levels of emotion.

Our multilevel model showed that, overall, youths with CD had higher FER biases than TDCs. While some studies have reported stronger anger biases in youths with CD [14, 34] and others have found no such effect [16], our findings therefore suggest that FER biases in CD extend beyond anger. In contrast to prior research highlighting increased biases for specific emotions - such as fear in the study by Short and colleagues [18] - youths with CD in our study exhibited stronger FER biases across all emotions. This is in line with FER accuracy research, showing that youths with CD perform worse in recognizing emotions in facial expressions [35]. In general, FER accuracy and FER biases overlap in the way that overall poorer emotion recognition accuracy also contributes to higher biases. However, FER accuracy alone does not indicate which incorrect emotions are chosen. For instance, youths with CD might show lower FER accuracy across all emotions but only exhibit a stronger bias toward anger. Alternatively, they could show lower FER accuracy across all emotions while having stronger biases for multiple emotions such as anger, sadness, and surprise. With our findings, we could show that lower FER accuracy in youths with CD is not driven by a bias toward a single emotion, but rather by generally increased FER biases. Due to the importance of FER for social interactions [4, 5], these findings seem to indicate a particular challenge in social situations for youths with CD. If these youths show increased tendencies to misinterpret emotional expressions, they are at risk to respond improperly to the other person and not react appropriately to a situation [5].

Our findings on FER biases also contribute to a novel understanding of how different emotions at different intensity levels are perceived: The difference between groups in FER biases did not vary with the type but rather with the intensity of emotions, such that the CD group had stronger biases at lower intensity levels compared to TDCs, and showed a smaller increase with increasing intensity level. It is theoretically expected that FER biases will get stronger as the intensity of the respective emotion increases, since higher intensity levels make emotional expressions clearer. The fact that the CD group showed a weaker increase in FER biases compared to TDCs and values below 50% for the 50% intensity level suggests that, under maximum ambiguity, they are more likely to select an emotion that is not present in the facial expression at all. While values below 50% for the 50% intensity level cannot be interpreted as a bias per se, the difference between youths with CD and TDCs at this intensity level might show that youths with CD make more random responses or errors. This pattern could, in turn, explain the higher FER biases of youths with CD observed at 0% intensity.

Our model showed a three-way interaction between group, emotion, and intensity level, indicating that group differences in FER biases across intensities varied by emotion. Notably, differences between youths with CD and TDCs in anger bias were more pronounced at the 10% and 30% intensity levels than at 0%. This is contradictory to previous research showing that, compared to other individuals with externalizing problems, such as ADHD, or CD and co-occurring ADHD, adolescents with CD only had a significantly stronger FER bias for the emotion anger when anger was not actually one of the expressions presented [12]. Additionally, the observed pattern in anger bias may reflect a heightened sensitivity to low-intensity representations of anger in youths with CD.

Our results contribute to and extend the sparse literature on CD and FER biases by incorporating emotion type as well as intensity level. This is important since investigating FER biases for all basic emotions might contribute to a better understanding of social behavior [6]. Furthermore, it might help to inform novel approaches for the treatment of CD by training emotion recognition skills with FER biases as treatment targets similar to Stoddard and colleagues [36]. These authors found that a computer-based training program, in which adolescents received positive feedback for rating ambiguous facial morphs as happy and negative feedback for rating them as angry, could shift anger biases toward more positive classifications of emotions. This training was also associated with reduced irritability and changes in brain activity in adolescents with Disruptive Mood Dysregulation Disorder (DMDD; which is characterized by frequent and intense anger and temper outbursts), indicating a potential novel treatment approach for CD youths with FER bias problems as well.

### Strengths and limitations

This study is the first to investigate FER biases in youths with CD for all six basic emotions and across different intensity levels. The use of low intensity and more ambiguous facial expressions reveals more subtle differences between groups [37] as these stimuli are also more sensitive to biases than prototypical emotional expressions [37]. In addition, multilevel models used are excellently suited to analyzing observations nested in individuals and investigating the influence of intensity and emotion type through their hierarchical structure [38]. Including group as a fixed effect and sex as a control variable in the final multilevel model ensures that observed group effects are not confounded by the differences between groups in sex distribution.

Despite the methodological strengths of this study, several limitations should be considered. First, the cross-sectional design of the study limits the ability to draw conclusions about the direction or existence of a causal relationship between group status (i.e., having CD) and FER biases. This leaves it unclear whether FER biases might serve as a predisposing risk factor for CD or whether CD itself might contribute to the development of FER biases. To examine whether FER biases predict future clinical outcomes (e.g., persistence or resistance of CD over time) or certain types of behaviors (e.g., aggressive symptoms), future longitudinal studies could measure changes in CD symptoms and FER biases over time. Our approach also does not fully capture the heterogeneity of CD and youths with CD showing different comorbidities, which could possibly influence FER biases. Furthermore, real-world social interactions involve dynamic, context-dependent emotional expressions, which are not fully captured in static FER tasks like the Emotion Hexagon Task. Future studies should consider incorporating more dynamic expressions, for example video-based, to improve ecological validity and better understand FER biases in CD.

Although our multilevel models accounted for individual differences and the hierarchical data structure, the inclusion of a random slope was not possible due to model convergence issues. This may limit the generalizability of the findings, as individual variations in response patterns could not be fully accounted for. Also, possible site effects were not included in the model. Adding a varying intercept did not lead to significant model improvement, which is surprising in view of the heterogeneity of neurocognitive functions in youths with CD [29]. Although significant, adding group as a fixed effect did not appear to make a large contribution to model improvement in both cases. Comparing the marginal variance for all multilevel models, the inclusion of emotion intensity seemed to improve model fit the most. Lastly, given the substantial overlap between accuracy and FER biases, we refrained from statistically controlling for overall accuracy to avoid removing meaningful variance from the bias scores. However, future studies might explore alternative methodological approaches to disentangle the influence of general accuracy on FER bias scores.

## Conclusion

To our knowledge, this study is the first to examine differences in FER biases across all basic emotions in youths with CD compared to TDCs. Our findings suggest that youths with CD show increased FER biases compared to TDCs irrespective of type of emotion. However, the strength of these differences changes with emotion intensity, with the CD group showing a smaller increase in FER biases as intensity increases. These results underscore the importance of further investigating the potential neuropsychological mechanisms underlying such FER biases in youths with CD and examining whether FER training can reduce FER biases in CD populations. Given that FER biases are linked to social difficulties and antisocial behavior, interventions aimed at improving specific emotion recognition skills are a valuable component of treatment approaches for CD.

## Supplementary Information

Below is the link to the electronic supplementary material.

Supplementary Material 1 (PDF 156KB)

## Data Availability

The data that support the findings of this study are not openly available. However, interested researchers can apply to the FemNAT-CD steering committee (chaired by C.M.F.) for data access as part of a collaboration.
