# Supplementary material for: Beyond hostility: exploring facial emotion recognition biases in youths with conduct disorder
Source: Eur Child Adolesc Psychiatry. 2025 Aug 27;35(1):253–62. doi: 10.1007/s00787-025-02846-y (PMC12917026; doi:10.1007/s00787-025-02846-y)
Supplement: Supplementary file 1 — Supplementary Material 1 (PDF 156KB) [file 787_2025_2846_MOESM1_ESM.pdf]

Supplementary Materials for "Beyond Hostility: Exploring Facial Emotion Recognition Biases in Youths with Conduct Disorder"

In: European Child & Adolescent Psychiatry

Janine Bacher, Beryll von Planta, Anka Bernhard, Graeme Fairchild, Lucres Jansen, Stephane A. De Brito, Christine M. Freitag, Kerstin Konrad, Christina Stadler, Gregor Kohls*, Eva Unternaehrer*

*These authors contributed equally to this work and share senior authorship.

Correspondence to:
Janine Bacher, Child and Adolescent Psychiatric Research Department, University Psychiatric Clinics Basel, Basel, Switzerland; e-mail: janine.bacher@upk.ch

Methods

Data Quality Criteria

We started cleaning our data by filtering the Emotion Hexagon Task data (total N = 1,549) to exclude participants who answered with the correct emotion in less than 20% of all trials. Since there is no right and wrong answer between two emotions with 50% intensity, rows were filtered out where this was the case, and one of the two emotions represented 50% in the face was contained in the participant's answer. This led to the exclusion of 10 participants. Next, we filtered out participants who answered with the same emotion in more than 50% of the trials, which led to the exclusion of 3 participants in total. In the next step, we excluded participants who answered with the same emotion consecutively 10 or more times, which led to the exclusion of 8 participants. After this step, we had a sample size of n = 1,528. Then, we combined the Emotion Hexagon Task data with the total femNAT-CD questionnaire data and included only those participants who participated in both the Hexagon Emotion Recognition Task and the cross-sectional questionnaire (n = 1,488). Lastly, we excluded those participants who fulfilled the exclusion criteria of IQ < 70 or had missing values for the group variable, which left us with a sample size of n = 1,430.

Data Analysis

R Packages

Data of the Emotion Hexagon Task were preprocessed using the dplyr R package to merge all individual data frames from the task into a single data frame (v1.1.4; Wickham et al., 2023a), the ggplot2 R package for visualization (v3.5.1; Wickham, 2016) and the gtools R package to combine multiple data frames with different column structures (v3.9.5; Warnes et al., 2023). To import the SPSS dataset with all questionnaire data, the haven R package (v2.5.4; Wickham et al., 2023b) was used. Internal consistency of questionnaire scales was calculated using the psych R package (v2.4.12; Revelle, 2024). Analyses of the multilevel models were conducted with the the tidyr R package for clean coding (v1.3.1; Wickham et al., 2024), the nlme R package to build hierarchical models (v3.1.166; Pinheiro et al., 2024) and the performance R package to check model fit (v0.13.0; Lüdecke et al., 2021).

Results

Supplementary Table 1

Comparison of Fixed Intercept and Random Intercept Models

	Model	AIC	BIC	logLik	Test	*p*	R^2
Fixed Intercept	1	305754.5	305771.4	-152875.2			
Random Intercept	2	305756.5	305781.8	-152875.2	1 vs 2	0.99	
Fixed Slope: Type of Emotion	3	305328.9	305396.5	-152656.5	2 vs 3	< 0.001	0.01
Fixed Slope: Intensity linear	4	285545.5	285579.3	-142768.8	2 vs 4	< 0.001	0.45
Fixed Slope: Intensity quad.	5	281347.8	281390	-140668.9	4 vs 5	< 0.001	0.51
Fixed Slope: Type + Intensity quad.	6	280459.8	280544.2	-140219.9	5 vs 6	< 0.001	0.52
Adjusted* Fixed Slope: Type + Intensity quad.	7	280360.6	280461.9	-140168.3	6 vs 7	< 0.001	0.52
Adjusted Fixed Slope + Group	8	280346.8	280456.6	-140160.4	7 vs 8	< 0.001	0.52
Adjusted Fixed Slope + Group + Group*Type	9	280338.7	280490.7	-140151.4	8 vs 9	0.003	0.52
Adjusted Fixed Slope + Group + Group*Type + Group*Intensity	10	280179.8	280348.6	-140069.9	9 vs 10	< 0.001	0.53
**Adjusted Fixed Slope + Group + Group*Type + Group*Intensity + Group*Type* Intensity**	**11**	**279378.8**	**279716.4**	**-139649.4**	**10 vs 11**	**< 0.001**	**0.54**
Adjusted Fixed Slope + Group + Group*Type + Group*Intensity + Group*Type* Intensity + Group*Age	12	279380.7	279726.8	-139649.3	11 vs 12	0.78	0.54
Adjusted Fixed Slope + Group + Group*Type + Group*Intensity + Group*Type* Intensity + Group*Sex	13	279380.5	279726.6	-139649.2	11 vs 13	0.6	0.54

*Notes. * Controlling for age and sex.* IC = Akaike's Information Criterion, BIC = Bayesian Information Criterion, logLik = log Likelihood, R^2 = marginal variance. **Bold** = This model explained the FER bias data best.

References

Lüdecke, D., Ben-Shachar, M. S., Patil, I., Waggoner, P., & Makowski, D. (2021). performance: An R package for assessment, comparison and testing of statistical models. Journal of Open Source Software, 6(60). 3139. https://doi.org/10.21105/joss.03139

Pinheiro J, Bates D, R Core Team (2024). _nlme: Linear and Nonlinear Mixed Effects Models_. R package version 3.1-166, <https://CRAN.R-project.org/package=nlme>.

Revelle, W. (2024). _psych: Procedures for Psychological, Psychometric, and Personality Research_. Northwestern University, Evanston, Illinois. R package version 2.4.12, <https://CRAN.R-project.org/package=psych>.

Warnes, G., Bolker, B., Lumley, T., Magnusson, A., Venables, B., Ryodan G, Moeller, S., (2023). _gtools: Various R Programming Tools_. R package version 3.9.5, <https://CRAN.R-project.org/package=gtools>.

Wickham, H., François, R., Henry, L., Müller, K., Vaughan, D. (2023a). _dplyr: A Grammar of Data Manipulation_. R package version 1.1.4, <https://CRAN.R-project.org/package=dplyr>.

Wickham, H., Miller, E., Smith, D. (2023b). _haven: Import and Export 'SPSS', 'Stata' and 'SAS' Files_. R package version 2.5.4, <https://CRAN.R-project.org/package=haven>.

Wickham, H. (2016). ggplot2: Elegant Graphics for Data Analysis. Springer-Verlag New York

Wickham, H., Vaughan, D., Girlich, M. (2024). _tidyr: Tidy Messy Data_. R package version 1.3.1, <https://CRAN.R-project.org/package=tidyr>.
